# Adaptive sampling methods facilitate the determination of reliable dataset sizes for evidence-based modeling

**DOI:** 10.3389/fbinf.2025.1528515

**Published:** 2025-09-04

**Authors:** Tim Breitenbach, Thomas Dandekar

**Affiliations:** Lehrstuhl für Bioinformatik, Biozentrum, Julius-Maximilians-Universität Würzburg, Würzburg, Germany

**Keywords:** model reliability, data size estimation, stochastic convergence to ground truth properties, stability of sampling properties, reliable alternative hypothesis formulation

## Abstract

How can we be sure that there is sufficient data for our model, such that the predictions remain reliable on unseen data and the conclusions drawn from the fitted model would not vary significantly when using a different sample of the same size? We answer these and related questions through a systematic approach that examines the data size and the corresponding gains in accuracy. Assuming the sample data are drawn from a data pool with no data drift, the law of large numbers ensures that a model converges to its ground truth accuracy. Our approach provides a heuristic method for investigating the speed of convergence with respect to the size of the data sample. This relationship is estimated using sampling methods, which introduces a variation in the convergence speed results across different runs. To stabilize results—so that conclusions do not depend on the run—and extract the most reliable information encoded in the available data regarding convergence speed, the presented method automatically determines a sufficient number of repetitions to reduce sampling deviations below a predefined threshold, thereby ensuring the reliability of conclusions about the required amount of data.

## Highlights

- We analyze the convergence speed of accuracies and uncertainties over data sample sizes for ML and mechanistic models.
- We develope an algorithm that stabilized statistic properties of distributions determined by repeated sampling.
- The approach is also applicable for estimating the data size at which test statistics exhibit sufficiently low variability, enabling the formulation of reliable hypotheses that do not depend on the concrete sampling.

## 1 Introduction

Assuming that measuring data is equivalent to randomly drawing samples from a data pool, a key aspect is determining the size of a sample dataset that sufficiently represents the properties of the data pool. According to the law of large numbers, model accuracy—or any other quantity, such as a test statistic—computed on a sample dataset converges to its ground truth value as the size of the data sample, which is used for model generation or calculating a test statistic, increases.

In related work, an optimal model size is investigated for a given dataset (Friedland et al., 2018). Moreover, how model size and data size are supposed to scale for a given compute budget is also analyzed (Hoffmann et al., 2022). Our investigation focuses on the analysis of how the accuracy of a model increases when the sample dataset increases while assuming a well-suited model for each sample set size. Mapping the model accuracy against the size of the dataset to estimate the dataset size at which a desired accuracy is achieved is called the learning curve approach and has already been explained in the following publications. One can increase the sample data size and monitor the accuracy of a trained model, and additionally monitor the spread of the accuracy given several randomly chosen samples from a pool for each sample size (Cortes et al., 1993; Morgan et al., 2003; Mukherjee et al., 2003; Figueroa et al., 2012). The presented work extends these works by introducing a mechanism that adaptively chooses the number of randomly chosen samples automatically for each sample size, thus stabilizing the statistical properties of the accuracy distributions on training and test sets. If the numbers for repetition are manually set too high, it might waste computational resources. If they are set too low, the results of the algorithm, such as the convergence speed and uncertainties, might change across different runs. By providing tolerances for the properties of the distributions, we directly control the allowable uncertainty based on the use case and avoid manually performing several runs to meet those tolerances. This approach is also computationally efficient as we perform only as many repetitions as needed to fulfill the required tolerances. The convergence of the presented procedure is grounded in mathematical theorems from probability theory. The convergence speed, which cannot be directly derived from such theory, might be influenced by the complexity of the dynamics generating the data, inherent noise (including that introduced by the measurement process and its inaccuracies), and model-specific factors such as the architecture and the training/fitting method. The purpose of our work is to provide a heuristic method, analogous to that of Mukherjee et al. (2003), for analyzing the convergence speed of the model with respect to accuracy as a function of sample size, its uncertainty decay, and for enabling predictions of these quantities for larger datasets; this method is extended with stabilized random sampling to produce reliable estimations of additional data in an automated and computationally cost-efficient manner. The overall benefit of using learning curve analysis, particularly in a production environment, is to obtain a model that meets certain quality and reliability requirements in terms of accuracy. This approach allows the prediction of expected accuracy and its associated uncertainty on unseen data, providing an important tool for ensuring quality.

A useful application of the presented framework arises in scenarios where data annotation in machine learning (ML) or data measurement in life sciences is expensive, and thus, the estimation of an optimal cost-to-reliability gain of a model is of importance, along with balancing its ratio when necessary.

We emphasize that the presented approach is not limited to ML models but works for any model, such as a mechanistic model (Mendoza and Xenarios, 2006; Breitenbach et al., 2021; Breitenbach et al., 2022), which can be represented by a function f mapping input variables x to output variables y . While in an ML scenario, x represents the values of the input features, in a mechanistic modeling scenario, x can be a vector of time, optionally space, and values of external stimuli (Breitenbach et al., 2019). In such a case, depending on the data model, y can be a measured data point or a best value (e.g., the mean) resulting from a repetition of the same experiment for the same values of x (Raue et al., 2013; Raue et al., 2015). An example of a mechanistic model is a system of ordinary differential equations (ODEs) where the function f is generated by solving the corresponding differential equations (Crouch et al., 2024). In such a case, the parameters of the ODEs have been fitted to the corresponding sample dataset such that x and y are best fit by f . These parameters are tested on the test set, consisting of data not used during model generation, to obtain the accuracy of the corresponding ODE solution f . Since time or space variables are often fixed (i.e., when and where to measure), collecting more data can be achieved by repeating the total experiment (e.g., to obtain other best values for the same values of x ), measuring over a longer time horizon or at more time steps or locations, or performing additional experiments with different external influences represented by the external stimuli, such as experiments with different intensities of the external influences or different combinations. In that case, the part of x representing time or space is fixed, and only the entries of x representing the external stimuli change.

A concept related to estimating the amount of data is the power estimation of a statistical test. The power of a statistical test is the probability of rejecting the null hypothesis if the alternative hypothesis is true. In the case of model fitting, the null hypothesis is that the model fits the data; thus, the model deviations from the data are only caused by random fluctuations, and a corresponding chi-square test of goodness-of-fit is used to test this hypothesis. The alternative hypothesis is that the model does not fit the data, and a non-central chi-square distribution can be used to calculate the probabilities of the observed chi-square values under the assumption that the alternative hypothesis is true. However, we need to know the expected deviation of the test statistic (how non-central the chi-square distribution should be) in advance. This estimation is carried out based on the available data and could vary depending on the specific data sample. Consequently, our proposed framework can also be used in a general manner to estimate required data size at which the estimated parameters of a statistical method, such as those used in power analysis or in determining the non-centrality parameter of a non-central chi-square test, vary sufficiently little; this allows for the formulation of a quantified alternative hypothesis with reliable parameter values, indicating that these values are sufficiently close to the ground truth values.

## 2 Methods

In this section, we present our method in Algorithm 1, which is based on Mukherjee et al. (2003), and explain it. A Python implementation of Algorithm 1 is provided in Supplementary Material.

In the following paragraphs, we explain Algorithm 1. In the first step, we set certain parameters. The parameter c determines how a data sample set is split into training and test sets. Analogous to the ML scenarios, a mechanistic model is fit on the training dataset, and its accuracy is also validated on the unseen test dataset. To obtain increasing data subsets sampled from the data pool D , which is the maximum data currently available, we randomly sample each subset of D according to some percentage numbers defined in the ordered set S where the percent values are in ascending order. The number m provides the number of parallel samplings to test whether the repetition numbers are large enough to ensure statistically stable properties. The larger the values of m , the more certain we can be that repeating the framework would yield the same result; however, the computational effort increases accordingly. The minimum value is 2 to compare at least two distributions of two different samples for a given sample size. The number $k^{0}$ is the minimum number of the repetitions of the sub-samplings drawn from D . This parameter should be large enough to ensure that the tests in Step 2.(d) are well-defined but not so large that the given tolerances are already satisfied as this could result in unnecessary computational effort. This second aspect is particularly important when model training is computationally expensive. The parameter $k_{n}$ is the corresponding repetition number for each n . The number $n_{0}$ is set higher than any number in S and represents the percentage of the data at which reasonable model learning starts, i.e., the point where the test set accuracy exceeds that of a model trained on a dataset where the output data is shuffled before training. Please see the explanation of the optional part of Step 2 later.

Algorithm 1 This algorithm identifies stable statistical properties of samples of different sizes to identify sufficiently large datasets on which a model has reliable properties.

1. Set the training and test data split 0<c<1 , an ordered set $S=:\left\{s\in R|\;0<s\leq 100\right\}$ , $k_{n}\leftarrow k^{0}\in N$ , n∈S , $N∋n_{0}>100$ , and 2≤m∈N .
2. For each n∈S , do:
    Repeat until BREAK, but at least $mk^{0}$ times:
   (a) Randomly draw n percent of samples from the pool dataset D , where the number of randomly drawn samples is denoted by $d_{n}\in N$ .
   (b) Split the randomly drawn data into training data with $cd_{n}$ samples and test data with $\left(1-c\right)d_{n}$ samples.
   (c) Generate the model and obtain the accuracies on the training set $a_{n,i,j}^{\text{tr}}\in R$ and the test set $a_{n,i,j}^{\text{te}}\in R$ , where $i\in \left\{1,\ldots ,k_{n}\right\}$ and $j\in \left\{1,\ldots ,m\right\}$ if no model has been created yet for the current i,j.
   (d) IF for each $\rho \in \left\{tr,te\right\}$ and for all $j_{1},j_{2}\in \left\{1,\ldots ,m\right\}$ , $j_{1}<j_{2}$ , the distributions $\left\{a_{n,i,j_{1}}^{\rho }|\;i\in \left\{1,\ldots ,k_{n}\right\}\right\}$ and $\left\{a_{n,i,j_{2}}^{\rho }|\;i\in \left\{1,\ldots ,k_{n}\right\}\right\}$ pass statistical similarity tests, then:
           BREAK the loop for the corresponding n .
        ELSE:
           Increase $k_{n}\in N$ and continue the loop getting the missing accuracies.
    Optionally:
     For each n∈S , if $n<n_{0}$ :
      Repeat steps 2.(a) to 2.(d) with the following changes:
       
• For Step 2.(c), generate a model on thetraining data where the ground truthoutput is shuffled, and obtain theaccuracies on the training set $b_{n,i,j}^{\text{tr}}\in R$ and the test set $b_{n,i,j}^{\text{te}}\in R$ , $i\in \left\{1,\ldots ,k_{n}\right\}$ ,
         and $j\in \left\{1,\ldots ,m\right\}$ if no model has been created yet for current i,j.
       
• Step 2.(d) is analog.
      If $\left\{a_{n,i,j}^{\text{te}}|\;i\in \left\{1,\ldots ,k_{n}\right\}\right\}$ and $\left\{b_{n,i,j}^{\text{te}}|\;i\in \left\{1,\ldots ,k_{n}\right\}\right\}$ are significantly distinct based on a statistical test for all $j\in \left\{1,\ldots ,m\right\}$ and the model on the non-shuffled data is more accurate, set $n_{0}\leftarrow n$ .
  3. For training and test sets, $\rho \in \left\{tr,te\right\}$ , fit a function $f^{\rho }:R\to R$ , $d\mapsto f\left(d\right)$ , with d representing the number of samples each mapping to model accuracy (or any other statistical property such as the percentile) based on the data points $\left\{\left(cd_{n},a_{n,i,j}^{\text{tr}}\right)|\;n\in S,i\in \left\{1,\ldots ,k_{n}\right\}\right\}$ or $\left\{\left(\left(1-c\right)d_{n},a_{n,i,j}^{\text{te}}\right)|\;n\in S,i\in \left\{1,\ldots ,k_{n}\right\}\right\}$ , respectively, for each $j\in \left\{1,\ldots ,m\right\}$ .
   Optionally: Exclude all data points with $n\geq n_{0}$ .
  4. Based on the fitted functions in Step 3 for the training and test sets, extrapolate the number of data points for the desired model accuracy or a desired tolerance of uncertainties.

The main idea of Step 2 is that we consider the dataset D as a pool from which we sample a corresponding n∈S percentage of data. Sampling only a percentage simulates the data collection process, where a measurement can be considered a random process, selecting a particular data sample from the ground truth data pool. Consequently, analogous to repeating the measurement process, our randomly repeated sampling from D results in different data subsets, which we use for model training and testing. The repetition is supposed to reveal the variance between different realizations of the sampling process. Depending on the sampling and the corresponding varying statistical properties of such randomly drawn sub-samplings, the model parameters and accuracy might be different. We know from theoretical results such as the Glivenko–Cantelli theorem or Donsker’s theorem (Billingsley, 2013; Durrett, 2019; Dudley, 2018) that the empirical cumulative density functions (CDFs) of D or any sub-sample of it converge to the corresponding ground truth CDFs as the number of measurements or dataset sizes increases, respectively. Analogously, we have the convergence of the probability density functions (PDFs) under certain conditions, such as in the kernel density estimator framework (Van der Vaart, 2000). Consequently, since the statistical properties of the sub-sampled datasets converge as their sizes increase, the properties of the subsets sampled from the data, such as the accuracy on the training and test sets, also converge under the assumption that models are continuous mappings with respect to their inputs and outputs. Identically, we have the convergence of the statistical properties of the m samples, meaning that they become more similar when increasing $k_{n}$ for each size of data subsets. Although we know about the existence of the convergence, we do not know how fast the convergence of the statistical properties of the datasets and the corresponding model properties takes place when increasing the number of samples in the data subsets. In Step 2.(c), we generate distributions of accuracies. In Step 2.(d), we compare the similarity of the statistical properties of these distributions. In our implementation, we test whether the corresponding pairs of mean, median, 25th percentile, and 75th percentile differ by less than a specified tolerance. These tests can be extended by any characteristic property of distributions, such as any stochastic moment, that allows defining a similarity measure of two distributions. Using statistical tests for defining significant similarity might be challenging as we need to execute them several times while increasing $k_{n}$ , and since it is not clear *a priori* how often we need to increase, a corresponding *p*-value adaptation to lower the error of the first kind upon many repetitions of the same test might not be possible. At the same time, introducing a tolerance parameter might be computationally beneficial as it eliminates the need to repeat sampling until the differences fall within the magnitude of random fluctuations but are small enough based on the use case requirements. In case the distributions are similar enough, we break. Otherwise, we repeat and extend the data foundation of the accuracy distribution for each j∈{1,…,m} while ensuring computational efficiency by keeping previous calculations. In our implementation, we increase $k_{n}$ by $k^{0}$ . However, any other strategy is possible, and increasing by a constant factor might be computationally more beneficial than a percentage increase, as, especially for bigger numbers, the increment scales exponentially, leading to large increments, which are not needed any more to achieve the desired tolerances. The model training in Step 2.(c) can (but does not have to) include hyperparameter tuning or any other operation to obtain a best fit of the model to the data. However, the method is equally valid when using fixed hyperparameters to investigate the corresponding convergence, and in a second run of the whole algorithm for a different set of fixed hyperparameters, one can examine whether and how these hyperparameters influence the convergence rate and the best achievable accuracy with large amounts of data. Particularly for mechanistic models, it can even be possible to change the ODE system and use a model that passes a chi-square goodness-of-fit test (Raue et al., 2013; Raue et al., 2015) for each fixed sample size. However, we can also use the same ODE system for all dataset sizes and just use some accuracy measure normalized to the number of data points, such as the $R^{2}$ -score.

The optional part of Step 2 is from Mukherjee et al. (2003) and is used to find the minimum sample size where the relations in the training data also hold mainly true on the test data. The core concept is to train a model on the training data, where the output is shuffled, and then to compare its prediction capability on a non-shuffled test set with a model trained on a non-shuffled training dataset. The aim of this procedure is to identify the minimum sample size at which relationships observed in the training set are also present in the test dataset, thereby ensuring a basic level of comparability in the statistical properties of the training and test data samples. We make sure that all distributions of accuracies are sufficiently stable before we compare the accuracies from the model trained on shuffled data with those of the model trained on non-shuffled data. In our implementation, we use the Mann–Whitney U test (scipy.stats.mannwhitneyu) to investigate the null hypothesis that both samples are drawn from the same distribution, where the *p*-value is calculated with regard to the alternative hypothesis that the model trained on the non-shuffled data has better accuracies on test data than the model trained on shuffled data. If the model cannot achieve better performance for larger sample sizes than the model based on shuffled data, it might be worth checking if the target is predictable at all given the input data (Zadeh et al., 2025).

In Step 3, for each $j\in \left\{1,\ldots ,m\right\}$ , we fit a function that models the convergence of the model accuracy vs data size on the training set $\left\{\left(cd_{n},a_{n,i,j}^{\text{tr}}\right)|\;n\in S,j\in \left\{1,\ldots ,k_{n}\right\}\right\}$ and the test set $\left\{\left(\left(1-c\right)d_{n},a_{n,i}^{\text{te}}\right)|\;n\in S,i\in \left\{1,\ldots ,k_{n}\right\}\right\}$ each. We assume a monotonic convergence. However, in general, which model best fits the convergence behavior is a model selection problem, as described by Figueroa et al. (2012). According to corresponding metrics, such as the Akaike information criterion (AIC) or the Bayesian information criterion (BIC), the model best fitting the data points can be chosen. There are different curve-fitting models available, as mentioned by Vianna et al. (2024); Curve fitting. In our case, as in the work of (Cortes et al.,1993; Morgan et al., 2003; Mukherjee et al., 2003, Cho et al., 2015), we choose a function based on the power law as follows:

$$f\left(d\right):=\alpha d^{\beta }+\gamma ,$$

where α,β,γ∈R are the optimization variables that are each fit for the training and test sets. We note that while the convergence of stochastic properties with increasing sample sizes is guaranteed to exist, there is generally no rule governing the specific behavior or rate of this convergence. In particular, it means that the convergence might not necessarily be a monotonic convergence. Consequently, just in case the convergence is (approximately) monotonic, the power law might be a good estimator for the limit of the convergence. However, in case of a non-monotonic convergence, plotting the accuracies with their uncertainties might provide helpful information about further potential of more data, the current state of uncertainty, or what function might be a good fit for the curve to make a well-fitting estimation regarding the limit accuracies and uncertainties. To maintain focus on how to adaptively and efficiently obtain stabilized distributions and thus stabilized optimization variables that ensure conclusions are not influenced by unfortunate sampling and remain comparable across different runs, we do not further discuss our model selection. We note that γ is the limit accuracy of d→∞ , which is the model accuracy the model approaches when increasing the data size of the data foundation, called the ground truth accuracy. In our implementation, for each n , we calculate the median and the mean as the best values and the 25th percentile and 75th percentile as measures of the variability in how the accuracies are distributed for the corresponding n . The calculations are based on $\left\{a_{n,i,j}^{\rho }|\;i\in \left\{1,\ldots ,k_{n}\right\}\right\}$ for each n∈S , $j\in \left\{1,\ldots ,m\right\}$ , and $\rho \in \left\{tr,te\right\}$ . We note that since these distributions might not be Gaussian, we instead use the percentiles as measures of the spread within $\left\{a_{n,i,j}^{\rho }|\;i\in \left\{1,\ldots ,k_{n}\right\}\right\}$ instead of the standard deviation. Based on the best values, we fit the curves according to ${\left\{\left(cd_{n},\text{mean}\left\{a_{n,i,j}^{\text{tr}}|\;i=1,\ldots ,k\right\}\right)\right\}}_{n\in S}$ and ${\left\{\left(\left(1-c\right)d_{n},\text{mean}\left\{a_{n,i,j}^{\text{te}}|\;i\in \left\{1,\ldots ,k_{n}\right\}\right\}\right)\right\}}_{n\in S}$ . Analogously, we calculate the data points for the median, 25th percentile, and 75th percentile. For the fitting, we weigh the residuals between the model and data points equally. The reason is that the uncertainties of all data points are, by construction, the same. In our implementation, we use a predefined tolerance for the absolute value of the corresponding differences between each pair of different $j\in \left\{1,\ldots ,m\right\}$ for n∈S during the similarity test. With that model, we can extrapolate to a data size that fulfills the requirements with respect to a sufficiently small uncertainty represented by the difference between the 75th and 25th percentiles or when the expected accuracy has sufficiently well-approached the ground truth accuracy. To fit the parameters, we use the curve_fit method from scipy.optimize in our implementation.

In step 4, we use the inverse of the fitted functions to extrapolate the amount of data that results in the desired accuracy and uncertainty requirements.

As already mentioned in the introduction, we can use Algorithm 1 to estimate the data size to obtain test statistics with a sufficiently small uncertainty, such as the non-centrality of the chi-square test or the effect of a therapy on shifting a distribution of a parameter in contrast to untreated patients. In case no model needs to be trained or parameters need to be fitted, such as the shift of a certain parameter under treatment, we modify Step 2.(b) in such a way that we split the randomly drawn data into m equally large sub-samples. Instead of the accuracy in Step 2.(c), we calculate the corresponding test statistic on each of the m sub-samples, such as the mean of a corresponding quantity, which then serves as the value of the parameters $a_{n,i,j}$ ; in this case, data are not split into training and testing subsets. By following the algorithm, we obtain a mean test statistic that accounts for the expected uncertainty and allows extrapolation to a dataset size where the uncertainty is sufficiently small. For example, in the case of treated and untreated patients, we need to perform the procedure for both populations. From the stabilized differences of the means of the test statistic, we can then calculate the effect strength for the hypothesis of the power analysis. Similarly, in an optimal experimental design, the sample size can be determined as the point at which the results of the corresponding analysis vary sufficiently little. Algorithm 1 can be used in cases where, instead of training a model in Step 2.(c), a corresponding analysis is performed and evaluated accordingly in Step 2.(d).

## 3 Results—showcasing the application of Algorithm 1

In this section, we showcase the application of Algorithm 1 based on the diabetes dataset from sklearn. The models are linear regression and the KNeighbors model from sklearn. To evaluate the success of the models, the $R^{2}$ -score for regression tasks from sklearn.metrics is utilized. sklearn’s standard scaler is fit on the training dataset and used to normalize the training and the test sets before each model training. For the split in Algorithm 1, the parameter c=0.7 . The hyperparameter n_neighbors = 25 for the KNeighborsRegressor if not otherwise stated. Furthermore, we choose m=2 and $k^{0}=300$ .

**FIGURE 1:**
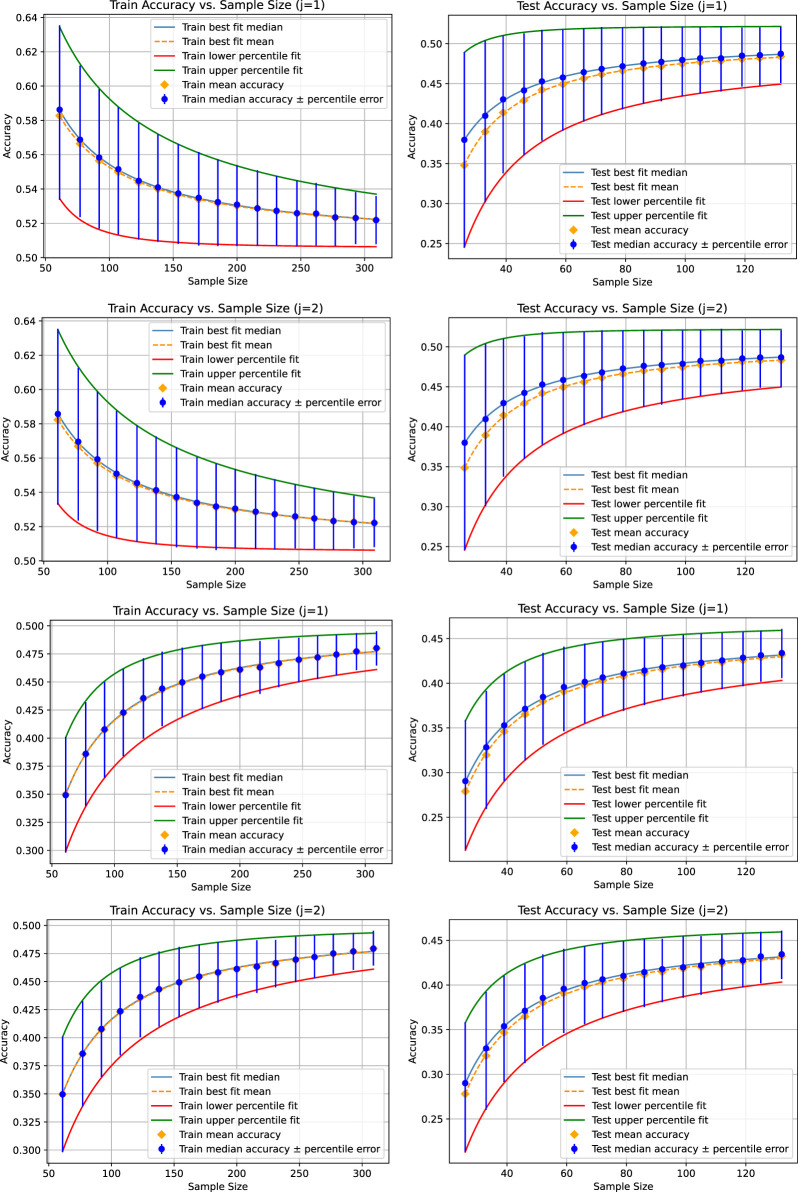
Results for the liner regression (top two figure rows) and the KNeighborsRegressor (bottom two figure rows) based on a tolerance of 0.001 for the absolute value of the difference between the mean, median, 25th percentile, and 75th percentile of each pair of accuracy distributions for each sample size. In this case, there are two instances of each accuracy distribution whose statistical characteristics are compared with respect to the tolerance, numbered by j=1 and j=2 ; see Algorithm 1 for the definition of j .

Figure 1 shows the fitted curves for the mean, median, and 25th and 75th percentiles for a tolerance of 0.001 for the absolute value of the difference between two means, medians, and 25th and 75th percentiles that we use to test similarity in Step 2.(d) of Algorithm 1 and the optional part of Step 2. The corresponding figures appear identical, demonstrating the function of the adaptive sampling mechanism. The limit accuracies are provided in Table 1.

**TABLE 1:** Mean value and standard deviation of the limit accuracy of the curves fitted to the means, medians, 25th percentile (25%-p), and 75th percentile (75%-p) of the accuracy distributions for all sub-sampling sizes. The first three rows are based on the training set, and the next three rows are based on the test set. The tolerance is the maximum number for the absolute value of the difference between two means, medians, and 25th and 75th percentiles. To calculate the mean and standard deviation for the limit accuracies, the results of three runs of Algorithm 1 are considered with m=2 ; thus, in total, six limit accuracies each are available. To put the numbers from the first six rows into perspective, we provide the results from the algorithms presented by Mukherjee et al. (2003) in the last two rows; this method uses a constant repetition number of 50 for each sample size to generate the accuracy distributions, which may appear intuitive, in the last two rows. In the second-to-last row, we provide the results based on the training dataset, and in the last row, the results are based on the test set. We note that the standard deviation is an order of magnitude larger as there is no control over the uncertainties in the sample distributions for each sample size.

Tolerance	Linear regression				KNeighborsRegressor			
	Mean	Median	25%-p	75%-p	Mean	Median	25%-p	75%-p
0.025	0.5125 ± 0.0025	0.5128 ± 0.0024	0.5061 ± 0.0012	0.4951 ± 0.0142	0.4992 ± 0.0049	0.4982 ± 0.0055	0.5060 ± 0.0110	0.5014 ± 0.0032
0.005	0.5092 ± 0.0009	0.5083 ± 0.0018	0.5060 ± 0.0005	0.4908 ± 0.0059	0.4985 ± 0.0013	0.4972 ± 0.0013	0.5097 ± 0.0040	0.5000 ± 0.0005
0.001	0.5084 ± 0.0007	0.5076 ± 0.0008	0.5057 ± 0.0002	0.4902 ± 0.0011	0.4984 ± 0.0004	0.4976 ± 0.0005	0.5084 ± 0.0011	0.4992 ± 0.0002
0.025	0.5037 ± 0.0081	0.5011 ± 0.0069	0.4887 ± 0.0100	0.5270 ± 0.0071	0.4543 ± 0.0073	0.4573 ± 0.0049	0.4547 ± 0.0197	0.4675 ± 0.0039
0.005	0.4993 ± 0.0022	0.5008 ± 0.0022	0.4860 ± 0.0035	0.5222 ± 0.0010	0.4531 ± 0.0026	0.4554 ± 0.0031	0.4536 ± 0.0061	0.4661 ± 0.0023
0.001	0.5006 ± 0.0007	0.5045 ± 0.0011	0.4890 ± 0.0021	0.5223 ± 0.0002	0.4544 ± 0.0004	0.4549 ± 0.0006	0.4540 ± 0.0014	0.4663 ± 0.0003
–	0.5110 ± 0.0076	0.5044 ± 0.0148	0.5057 ± 0.0033	0.4510 ± 0.0894	0.4951 ± 0.0097	0.4922 ± 0.0104	0.5123 ± 0.0334	0.4995 ± 0.0053
–	0.5116 ± 0.0283	0.5172 ± 0.0271	0.5153 ± 0.0530	0.5218 ± 0.0049	0.4737 ± 0.0431	0.4517 ± 0.0182	0.4932 ± 0.0853	0.4745 ± 0.0163

**FIGURE 2:**
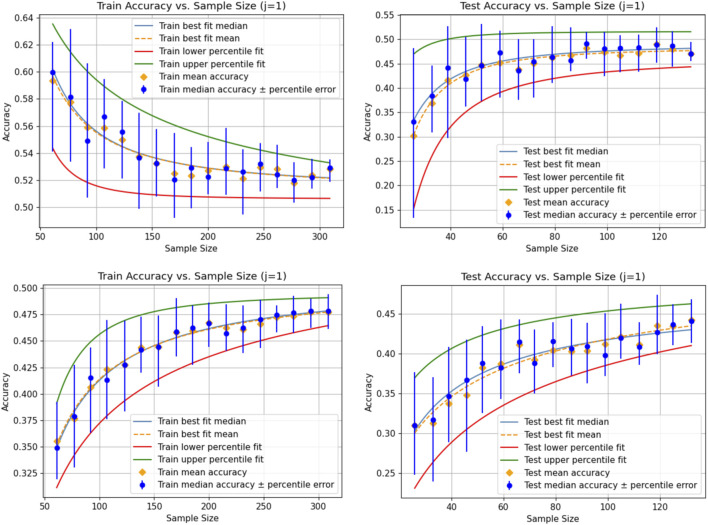
Results similar to Figure 2; however, the accuracy distributions for each sample size are generated according to Mukherjee et al. (2003) with a sampling number of 50 per distribution. In the first row, we see the result for the linear regression model, and in the second row, we see the result for the KNeighborsRegressor model.

The accuracy distributions underlying the graphs of Figure 2 are calculated according to the method described by Mukherjee et al. (2003) with a sampling number of 50 data points per distribution. We observe a higher variation in the statistical characteristics of the distributions for each sampling size compared to Figure 1. A larger number of data points will provide less variation; however, it is challenging to estimate a fitting number *a priori*. Our method automatically finds a suitable number of data points such that the variation is below a certain tolerance, which is even adaptive to each sampling size.

In Table 1, we compare the limit accuracy of the curves fitted to the four statistical characteristics of the accuracy distributions over the sample size with respect to their variation across different runs of our algorithm. To illustrate this, we choose to calculate the mean and standard deviation (square root of the standard variance) based on three runs of Algorithm 1 to observe how this variation decreases if tolerances for the differences of statistical characteristics decrease. Since m=2 , there is one repetition of the sampling of the accuracy distributions such that there are two instances of the four characteristics for each sample size, numbered j=1 and j=2 ; see Algorithm 1 for a definition of j . We note that the parameters of the fitted curves become more reliable as the tolerance decreases, showcasing that our proposed method for adaptively determining repetition parameters to achieve stable results works as expected.

Since the model with its architecture and hyperparameter setting is a part of the convergence process and thus influences the amount of data needed for stable and reliable results, we demonstrate in Table 2 how our proposed method can reveal that overfitting might be due to an unfortunate hyperparameter setting ( k representing n_neighbors of the KNeighborsRegressor) rather than an unfortunate sampling run. An unfortunate parameter setting occurs when the limiting accuracies are stable across different j instances, which numbers the repetitions used to generate accuracy distributions to test for statistical stability, yet a gap between the training and test limiting accuracies remains. If the limit accuracies from fitted curves based on stable statistical properties differ up to a degree that is considered too much, it is a hint that more data in the current model configuration might not resolve the overfitting but rather a hyperparameter change as we observe that a higher k lowers the gap. For such experiments to make such investigations in a reliable manner, the control over the uncertainty of the important characteristics of the samples is very helpful. The presented adaptive schema ensures reliability with a tailored repetition number. However, with respect to overfitting, linear regression might be a good choice in the presented example as it has a higher limit accuracy and a smaller gap between the training and test limit accuracy (Table 1). Another effect regarding overfitting can be observed in Figure 1. As the number of weights of the linear regression model is fixed for any sample size, we observe the effect of overfitting, indicating that the model adapts too much to the training dataset if the number of data points of the training data is smaller than the number of weights. Since the weights allow the model to closely fit its output to the small number of data points, including the noise, it leads to high accuracy on the training dataset. However, due to potential variance between the training and test sets, caused either by the small sample size or the presence of noise, the relationships learned from the training set do not generalize in detail to the test dataset, leading to poor accuracy on the test set. By increasing the sample size, the training and test sets converge in their statistical properties, and if the number of data points gets bigger than the number of weights, the model cannot adapt to any small variance in the training dataset but has to focus on the main relations (which means being supported by many data samples where a specific noise relation would only hold for a specific data point) that generally also apply on the test set. The result is that the accuracy gap between the training and the test sets decreases as the data size increases. Consequently, in case of linear regression, the accuracy on the training data decreases with the sample size, as it cannot adapt to any specific relation on the training data. At the same time, as the sample size increases, more of the relations in the training data are likely contained in the test data as well, while the model is not deflected by learned relations that only hold on the training set, causing increasing accuracy.

**TABLE 2:** Limit accuracy on the training (Train) and test (Test) sets of the curves, each fitted for the mean and median. The difference between the training and test limit accuracy is provided in the column named “Diff” for different k , modeling the number of neighbors (n_neighbors), and j , which numbers the instances of accuracy distributions; curves are fitted to the characteristics (mean and median) of these distributions, from which limit values are derived.

k	Type	j = 1			j = 2		
		Train	Test	Diff	Train	Test	Diff
10	Mean	0.5603	0.4709	0.0894	0.5605	0.4718	0.0887
	Median	0.5572	0.4773	0.0799	0.5581	0.4787	0.0794
30	Mean	0.4906	0.4547	0.0359	0.4910	0.4557	0.0353
	Median	0.4899	0.4564	0.0335	0.4895	0.4558	0.0337
50	Mean	0.4849	0.4615	0.0234	0.4847	0.4617	0.0230
	Median	0.4854	0.4671	0.0183	0.4850	0.4672	0.0178

**FIGURE 3:**
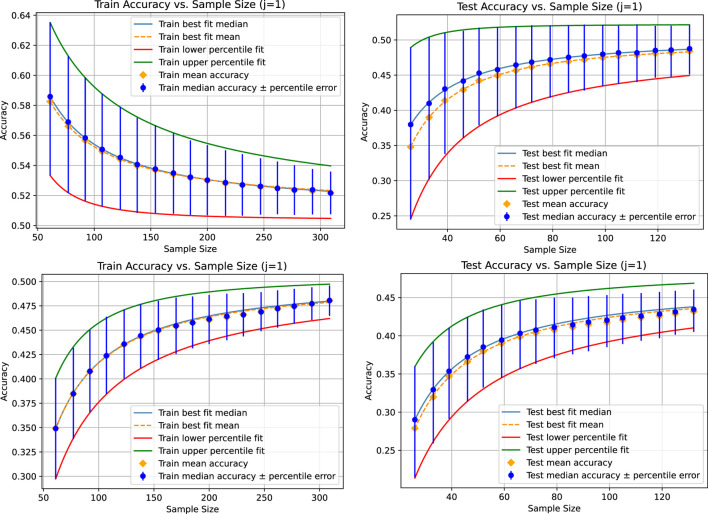
Results for the linear regression (top) and the KNeighborsRegressor (bottom) based on a tolerance of 0.001 for the absolute value of the difference between the mean, median, and 25th and 75th percentiles of the accuracy distribution for each sampling size compared pairwise. In this case, there are two accuracy distributions for each sampling size, each denoted with j=1 and j=2 ; see Algorithm 1 for the definition of j . The curves are fitted only on the first seven data points. For the linear regression, the limit accuracies (train/test) are 0.5101/0.4966 for the median, 0.5118/0.4966 for the mean, 0.5038/0.4780 for the 25th percentile, and 0.5038/0.5243 for the 75th percentile. For the KNeighborsRegressor, the limit accuracies (train/test) are 0.5023/0.4690 for the median, 0.5004/0.4683 for the mean, 0.5057/0.4778 for the 25th percentile, and 0.5053/0.4842 for the 75th percentile.

In the next experiment, we fit the curves only to the first seven data points and compare how the model predicts the ones not fitted to. Figure 3 shows the results. The points not used for fitting the power law can be used as a measure of goodness-of-fit and can also be used for model selection, helping identify which model best predicts the limit convergence behavior. This experiment is intended to show that the power law might only be an approximation for the convergence behavior. For example, the fitted parameters might only be valid up to a certain sample size. When using the curve to estimate the additional amount of data to reach certain accuracy gains or levels of uncertainty, we can use sanity checks to determine whether the model might still be valid for the sample size we predict. For example, if the curves for the 25th and 75th percentiles already intersect before the estimated data size. An intersection occurs for the example shown in Table 1 because the limit accuracy for the 25th percentile is higher than that for the 75th percentile on the training set. However, on the test set, the limit accuracies are in the right order. At the latest, after the intersection of the two curves, we would know that we are outside the area where such fitted curves might reliably be used. If our data requirements are not fulfilled before that point and other models do not fit better, meaning that they do not exhibit the same issue, this would be a strong indication that more data must be collected before repeating the analysis. With our proposed method, we can ensure that the identified area where the model is no longer reliable is not due to an unfortunate sampling but is a stable pattern from which we can draw corresponding conclusions.

## 4 Discussion

This work aims to provide a heuristic method to analyze the convergence speed of a model to the achievable ground truth accuracy when increasing the size of the data, reducing the effects of sampling variations by introducing tolerances for the corresponding distribution differences. By repeatedly sampling from the available data pool—starting with small amounts of data and gradually increasing the sample size, where each set represents one realization of a potential measurement of that size—we can observe the convergence behavior. Furthermore, we can observe how the uncertainty of the accuracy decreases to a certain range. This is particularly relevant when the reliability of a model in production is important as we can estimate how the accuracy might vary on the unseen data and, thus, whether the model is applicable at all or what amount of training data might still be needed. An example might be personalized medicine. The presented method is general and applicable to all types of models, whether ML or mechanistic.

Increasing the size of the dataset captures more of the underlying dynamics involved in data generation. If the dataset is large enough, there is a high probability that it contains a sufficient number of instances to learn all relevant aspects of these dynamics, regardless of the sampling. By providing broad information about the process to be modeled, we ensure that modeling is not overly specific to a dataset and that the model captures the ground truth dynamics, allowing it to perform reliably in real-world use cases. To apply the presented framework and deal with the many model trainings/fittings that are needed, the total process of model training/fitting needs to be automated to lower the costs of this procedure, e.g., for manual work. We need to run our presented method initially from a data pool D that is large enough to sample smaller subsets where the models can be properly trained/fitted. Additionally, the data pool D should already be diverse enough to capture different effects of the underlying dynamics; otherwise, consistently high accuracy might be observed across all sizes of sub-samplings, which would be misleading.

Estimating when reaching a certain degree of reliability in the accuracy is particularly important when data acquisition involves high costs. Examples are in life sciences when working with samples from patients or other samples whose preparation needs a lot of manual work or when there are costly annotation processes such as in ML scenarios, e.g., in natural language processing. With the suggested framework, realistic budgets for data acquisition can be estimated to bring a project to production. Having a clear view of the required data after a proof-of-concept might be crucial to estimating the remaining costs to bring a model to the desired reliability and quality required for production.

One limitation of the framework is that a model needs to be trained several times, which can become an issue in terms of computational costs for large models, such as language models. However, we note that training from scratch might not always be needed as a trained/fitted model from previous data samplings can be used, and the parameters can just be fine-tuned on other datasets to the corresponding parameters optimal for the specific sampled dataset. We note that an implementation of Algorithm 1 can be highly parallelized, e.g., for each n calculating batches of model training for different splittings in Step 2.(c). When a model is computationally too costly to execute the proposed method for different splittings for different n , we can validate the given data by training and testing a model on different splittings (cross-validation) of the whole data to obtain a distribution of accuracies on the training and test data. Then, we can see the corresponding uncertainties of the accuracies and report them or decide whether the uncertainty is sufficiently small.

Furthermore, in the case of estimating whether the collection of new data for an existing dataset is required to provide greater model reliability, we assume that the data quality stays the same over time. In particular, we assume that there is no drift in the data as the achievable ground truth accuracy might change because of more noise or data drift. In such a case where the ground truth dynamic changes rapidly compared to the data collection speed, an overall convergence is not guaranteed. An example of changing dynamics can be found in time-series prediction, particularly in predicting pandemic evolution (Vianna et al., 2024), where abrupt and unpredictable changes—such as new rules established by governments—can significantly impact outcomes*.* For time-series prediction, due to the issue that we cannot interchange data between the training and test sets (it would represent bringing information from the future to the present and thus obscure an ongoing dynamic change), we cannot directly apply our proposed method. As proposed by Vianna et al. (2024) (Figure 1), we can extend the time horizon defining the present to simulate obtaining more historical data for training while keeping the future data regarding the current present (or only a fixed period into the future) for validation to monitor the convergence of the model. However, if we assume that there is no dynamic change, such as in data from a periodic process (like the orbits of planets), then our method can be applied to estimate the convergence of the model with respect to the data sampling size. Although it is a time-series prediction, as there is no dynamic change, the system is closed without absolute time, and the temporal order is only important within the features of a model but not between different data points.

Our framework can be applied in cases where studies claim that an insufficient amount of data is a limiting factor (Hwang et al., 2024; Rodrigues, 2019; Sapoval et al., 2022; Ching et al., 2018). It can be used to estimate the approximate amount of additional data needed, allowing for more accurate planning of the costs of further studies, particularly the costs for data acquisition. This is similar to the approach of Valeri et al. (2023), where it was tested *a posteriori* whether less amount of data generation would be sufficient in subsequent experiments. Another application of our framework is where models are used to approximate computationally costly functions, such as mutual information between many random variables (Franzese et al., 2024). Mutual information describes how much knowing the value of one random variable reduces the uncertainty about another, i.e., how dependent the variables are on each other in their value distributions. Although such functions might be approximations themselves, which might cause a deviation from the ground truth mutual information, our method deals with estimating the amount of data needed to avoid additional variation in accuracy caused by an insufficiently small data sample. In this case, the approximated value does not depend on the size of the data but only on the mechanism of the approximation. However, in the case of convergence of the approximation to the ground truth mutual information for increasing data, our framework also includes an estimation of the speed of this convergence as our framework considers all components involved in the modeling of the data. Furthermore, with our presented framework, we can study models differing in the hyperparameter setting and test which model converges to a better limit accuracy or which is faster in convergence, similar to Valeri et al. (2023) (Supplementary Figure S1), where different models perform differently well for small datasets. One example can be a model with more layers or free parameters, which tests whether the smaller model is too small, and the other bigger model can store more of the information, which might be the case if the model with more free parameters leads to better limit accuracies on the training and test sets. In case both models converge to the same limiting accuracy, the smaller model is preferable and seems sufficient to represent the data and underlying dynamic.

## Data Availability

Publicly available datasets were analyzed in this study. This data can be found here: sklearn diabetes.
